# Childhood Trauma, Emotional Awareness, and Neural Correlates of Long-Term Nicotine Smoking

**DOI:** 10.1001/jamanetworkopen.2023.51132

**Published:** 2024-01-11

**Authors:** Annika Quam, Kathryn Biernacki, Thomas J. Ross, Betty Jo Salmeron, Amy C. Janes

**Affiliations:** 1 Neuroimaging Research Branch, National Institute on Drug Abuse Intramural Research Program, National Institutes of Health, Baltimore, Maryland

## Abstract

**Importance:**

Temporal dynamic measures provide insight into the neurobiological properties of nicotine use. It is critical to determine whether brain-based measures are associated with substance use risk factors, such as childhood trauma–related emotion dysregulation.

**Objective:**

To assess temporal dynamic differences based on smoking status and examine the associations between childhood trauma, alexithymia, nicotine smoking, and default mode network (DMN) states.

**Design, Setting, and Participants:**

This cross-sectional study was conducted in the Baltimore, Maryland, area at the National Institute on Drug Abuse. Participants included individuals aged 18 to 65 years who smoked nicotine long term and matched controls with no co-occurring substance use or psychiatric disorders. Participants were enrolled from August 8, 2013, to August 9, 2022. Analysis was conducted from August 2022 to July 2023.

**Exposure:**

Long-term nicotine smoking.

**Main Outcomes and Measures:**

The main outcome was temporal dynamic differences based on smoking status. Coactivation pattern analysis was conducted based on 16-minute resting-state functional magnetic resonance imaging; total time in, persistence of, and frequency of transitions into states were evaluated. The associations between childhood trauma (Childhood Trauma Questionnaire), alexithymia (20-item Toronto Alexithymia Scale), and DMN temporal dynamics were assessed.

**Results:**

The sample included 204 participants (102 individuals who smoked nicotine and 102 control individuals) with a mean (SD) age of 37.53 (10.64) years (109 [53.4%] male). Compared with controls, individuals who smoked nicotine spent more time in the frontoinsular DMN (FI-DMN) state (mean difference, 25.63 seconds; 95% CI, 8.05-43.20 seconds; η^2^*_p_* = 0.04; *P* = .004 after Bonferroni correction). In those who smoked nicotine, greater alexithymia was associated with less time spent in the FI-DMN state (*r*, −0.26; 95% CI, −0.44 to −0.07; *P* = .007). In a moderated mediation analysis, alexithymia mediated the association between childhood trauma and time spent in the FI-DMN state only in individuals who smoked nicotine (*c*′ = −0.24; 95% CI, −0.58 to −0.03; *P* = .02).

**Conclusions and Relevance:**

Compared with controls, individuals who smoked nicotine spent more time in the FI-DMN state. Among those who smoked nicotine, childhood trauma–related alexithymia was associated with less time spent in the FI-DMN state, indicating that considering trauma-related factors may reveal alternative neurobiological underpinnings of substance use. These data may aid in reconciling contradictory findings in prior literature regarding the role of FI-DMN regions in substance use.

## Key Points

**Question:**

Is there a neurobiological association between childhood trauma, alexithymia, and long-term nicotine smoking?

**Findings:**

In this cross-sectional study of 102 individuals who smoked nicotine long term matched with 102 healthy controls, individuals who smoked nicotine spent more time in the frontoinsular default mode brain network. Alexithymia mediated the association between childhood trauma and time spent in the frontoinsular default mode network only in individuals who smoked nicotine.

**Meaning:**

The findings suggest that distinct neurobiological profiles noted in those who smoke nicotine are associated with childhood trauma–related alexithymia.

## Teaser

This cross-sectional study examines differences in temporal dynamic brain states as a function of long-term nicotine smoking and associations of childhood trauma, alexithymia, and smoking with brain states.

## Introduction

The neurobiological mechanisms of long-term tobacco use have generated significant interest due to the substantial harm posed by smoking.^1,2^ Clinically, functional magnetic resonance imaging (fMRI) has identified ways in which tobacco use is associated with brain function, including inherent resting-state functional organization.^3^ Such investigations have primarily centered on static connectivity measures, which calculate correlated brain activity over time.^4^ In contrast, emerging evidence indicates that dynamic resting state analyses capture time-varying or dynamic patterns of functional coordination among brain systems,^5^ providing insight into the essential features of large-scale network function^6^ underlying cognition and psychopathology.^7,8^ Thus far, to our knowledge, there has been no evaluation into how temporal dynamic profiles differ between those who do and do not smoke, nor is there evidence indicating how smoking-related risk factors, such as emotional dysregulation or childhood trauma, influence temporal dynamics.

Using data from the Human Connectome Project (HCP), Janes et al^9^ conducted a coactivation pattern (CAP) analysis with a temporal dynamic approach^10,11^ to identify 8 transient network states. These states overlap with core neurocognitive networks, including the default mode network (DMN) and salience network (SN), which have been previously implicated in substance use disorders.^12,13^ Specifically, elements of the DMN and SN reliably react to smoking-associated cues.^14^ Moreover, communication between these networks is enhanced during smoking withdrawal,^15^ and greater DMN-SN connectivity has been associated with more craving.^14,16^

Temporal dynamics offer another means to explore the function of such substance use disorder–related networks. A temporal dynamics study in healthy individuals^9^ defined 3 DMN-related states: the frontoinsular DMN (FI-DMN), the canonical DMN, and the occipital sensorimotor DMN (DMN-OSM). That study proposed that the FI-DMN and DMN-OSM states share functions similar to the canonical DMN state, such as supporting self-referential thought, but that the type of self-referential processing may differ based on the other regions coactivating with typical DMN regions.^9^ For example, the FI-DMN state may involve more affective or salience aspects of self-referential processing, such as recalling an emotional event, since this state includes SN regions such as the insula. The association between the FI-DMN state and emotional processing was supported by Kaiser et al,^7^ who showed that spending more time in the FI-DMN state corresponded with greater ruminative focus on negative feelings.

Given the role of the DMN, SN, and FI-DMN states in emotion,^17,18^ it is tempting to speculate that the temporal dynamics of such states may be related to clinical measures of emotion dysregulation, such as alexithymia, which is characterized by difficulty identifying or describing feelings and by externally oriented thinking.^19^ Alexithymia is not only greater in those who do vs do not smoke^20,21^ but is also a risk factor for substance use and other psychiatric issues.^22,23^ Furthermore, alexithymia is associated with neurobiological variation in substance-using individuals but not in healthy controls,^12,24^ suggesting that considering alexithymia when measuring temporal dynamics may further clarify individual profiles, particularly in individuals who smoke nicotine long term. Childhood trauma is another related clinical factor to consider, as maltreatment has been shown to neurobiologically differentiate individuals with the same psychiatric diagnosis.^25^ There is substantial evidence not only that alexithymia is precipitated by childhood trauma^26^ but also that alexithymia mediates the association between childhood trauma and other issues, such as adult psychopathology,^27^ self-injurious behavior,^28^ and vaping.^29^ Thus, it is critical to determine whether alexithymia mediates the association between childhood trauma and temporal dynamics.

Collectively, the current work aimed to assess temporal dynamic differences between those who do and do not smoke nicotine, considering all 8 brain states previously defined using HCP data.^9^ This fills a gap in the field of neuroimaging and substance use pertaining to the general understanding of how temporal dynamic brain properties differ as a function of long-term nicotine smoking. Furthermore, the present study investigated the association of childhood trauma, alexithymia, and tobacco smoking with DMN networks.

## Methods

### Participants

In this cross-sectional study, data on individuals who smoked nicotine long term and nonsmoking healthy control individuals were taken from a larger cohort of an ongoing study conducted at the National Institute on Drug Abuse (NIDA) in the Baltimore, Maryland, area. The current study was reviewed and approved by the institutional review board of the National Institutes of Health; all procedures contributing to this work complied with the ethical standards of the relevant national and institutional committees on human experimentation and with the Declaration of Helsinki,^30^ as revised in 2008. Written informed consent was obtained from all participants. We followed the Strengthening the Reporting of Observational Studies in Epidemiology (STROBE) reporting guideline.^31^

Participants aged 18 to 65 years were enrolled from August 8, 2013, to August 9, 2022. Control individuals were best matched to individuals who smoked nicotine long term based on sex and age using the matching package, version 4.10-8, in R, version 4.3.0 (R Project for Statistical Computing); however, a significant group difference in age remained after matching (Table).^32^ Age was controlled for in all analyses. Race and ethnicity were ascertained by self-report and reported to ensure a representative sample of the Baltimore area; race categories were African American or Black, Asian, White, and multiracial, and ethnicity categories were Hispanic, non-Hispanic, and unknown or unreported. Controls had not used nicotine products within the past 12 months and had fewer than 10 lifetime uses. Individuals in the nicotine group had smoked cigarettes for at least 1 year prior to study participation, as confirmed by expired carbon monoxide. Regular smoking patterns were maintained prior to scanning. All participants had no co-occurring substance or alcohol use disorders, neurological disorders, or current major mood, anxiety, or psychotic disorders as assessed by the *Diagnostic and Statistical Manual of Mental Disorders* (Fourth Edition)^33^ or *Diagnostic and Statistical Manual of Mental Disorders* (Fifth Edition).^34^ All participants tested negative for current drug use, and females tested negative for pregnancy. Eligibility criteria for the larger NIDA parent study included other substance use, as that study was designed to obtain a common set of characteristics in participants enrolled in other studies at the NIDA Intramural Research Program. However, across all participants included in the parent study, inclusion and exclusion criteria were the same as described herein.

**Table.** Demographic and Smoking Characteristics

Baseline characteristic	Participants^a^		Group statistic	*P* value
	Nicotine (n = 102)	Control (n = 102)		
Age, mean (SD), y	39.32 (11.25)	35.75 (9.73)	*t* = 2.43	.02
Sex				
Female	47 (46.1)	48 (47.1)	χ^2^_1_ = 0.02	.89
Male	55 (53.9)	54 (52.9)		
Race				
African American or Black	55 (53.9)	22 (21.6)	NA	.001^b^
Asian	0	11 (10.8)		
White	41 (40.2)	63 (61.8)		
Multiracial	6 (5.9)	6 (5.9)		
Ethnicity				
Hispanic	3 (2.9)	5 (4.9)	NA	.46^b^
Non-Hispanic	99 (97.1)	95 (93.1)		
Unknown or not reported	0	2 (2.0)		
Educational level				
Less than high school	14 (13.7)	1 (1.0)	NA	.001^c^
High school completed or GED	37 (36.3)	19 (18.6)		
Some post–high school	34 (33.3)	30 (29.4)		
College graduate or bachelor’s degree	13 (12.7)	29 (28.4)		
Master’s degree	3 (2.9)	18 (17.6)		
Professional degree—MD, JD, PhD	1 (1.0)	5 (4.9)		
Substance use				
Nicotine dependence, mean (SD)^d^	4.65 (2.16)	NA	NA	NA
Carbon monoxide level, mean (SD), ppm	17.82 (9.06)	1.36 (0.78)		
Cigarettes smoked per day, mean (SD), No.	16.24 (9.34)	NA		

Abbreviations: GED, general educational development; NA, not applicable.

^a^ Data are presented as the number (percentage) of participants unless otherwise indicated.

^b^ Fisher exact test.

^c^ Ordinal regression.

^d^ Based on the Fagerström Test for Nicotine Dependence.^32^

### fMRI Acquisition and Data Preprocessing

Data were acquired using a 32-channel head coil on either a 3T Trio (Siemens Healthineers) or Prisma (Siemens Healthineers) MRI scanner during a 16-minute resting-state scan. Scanner type was controlled for in all analyses. Scan data were preprocessed using fmriprep, version 20.2.7^35^ including corrections for automatic removal of motion artifacts. The eAppendix in Supplement 1 provides scan parameters and preprocessing information.

### Resting-State Coactivation Pattern Analysis

**Figure 1.**
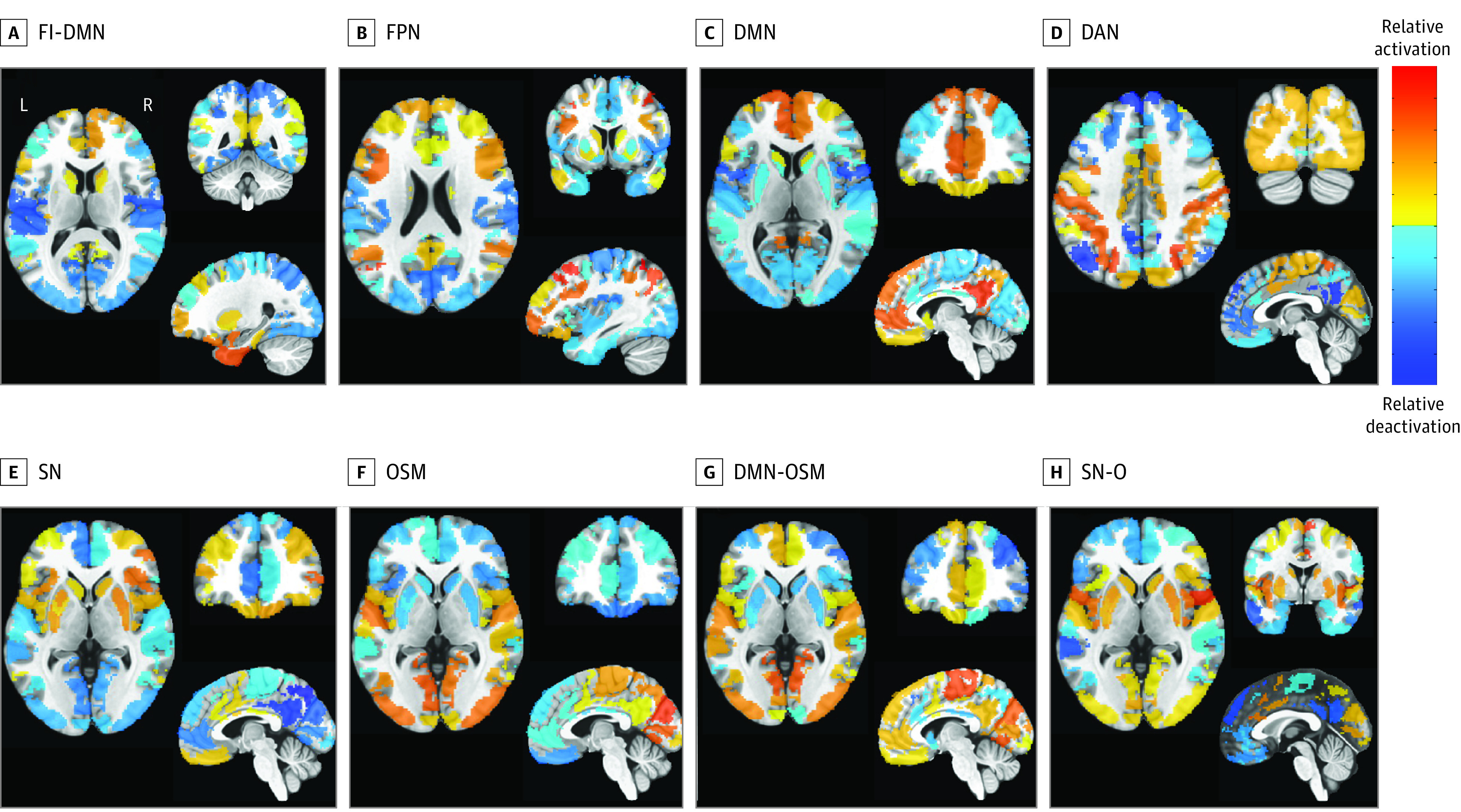
Coactivation Patterns Visualized in the Montreal Neurological Institute (MNI) Space Normalized coactivation patterns for each state used cluster centers from Janes et al^9^ applied to participants. The *x*, *y*, and *z* MNI coordinates for each state were as follows: frontoinsular default mode network (FI-DMN), −26, −48, and 12; frontoparietal network (FPN), −38, 8, and 22; default mode network (DMN), −6, 44, and 6; dorsal attention network (DAN), −2, −86, and 42; salience network (SN) 1 and occipital sensory-motor (OSM), −4, 46, and 0; DMN-OSM, −6, 44, and 0; and SN-O, 0, 0, and 0.

We used the 8 whole-brain CAPs extracted by Janes et al.^9^ These CAPs were derived using 129 regions of interest and *k*-means clustering on resting-state data from 462 individuals from the HCP. Principal components analysis preprocessing and CAP analyses were conducted using the capcalc package, version 1.3.5^36^ (Figure 1 and eFigure 1 in Supplement 1). The CAP parameters extracted were time in state (total scan time spent in a state), persistence in state (mean time spent in a state per entry), and proportion of transitions to state (number of transitions to a state divided by total number of transitions).

### Childhood Trauma and Trait Alexithymia Assessment

Childhood trauma was assessed using the Childhood Trauma Questionnaire (CTQ).^37^ Alexithymia was assessed using the 20-item Toronto Alexithymia Scale (TAS-20).^19^

### Statistical Analysis

#### Group Differences in Dynamic Brain States

Data were analyzed from August 2022 to July 2023. Group comparisons of time in brain states were conducted in SPSS, version 29 (IBM Corp). Separate repeated-measures analyses of variance (ANOVAs) evaluated the total time in state and persistence in state. Each ANOVA considered a main effect of group (nicotine, control), state (8 CAP networks), and their interaction and controlled for scanner type. Two-proportion *z*-tests were applied using the stats package, version 3.6.2, in R, version 4.5.0, to assess any group differences in transitions to state given that the total number of transitions to each state in each group differed. All post hoc tests were corrected using the Bonferroni correction. Any analyses that violated assumptions of sphericity used the Greenhouse-Geisser correction. The Greenhouse-Geisser correction is conservative and was used because the estimates of sphericity were small. All hypothesis tests were 2-tailed.

#### Interactions Between Alexithymia, Childhood Trauma, and Brain State

Two separate linear regressions compared the association of CTQ or alexithymia with group and brain state and their interactions with total time in state using the car package, version 3.1-2, in R, version 4.3.0. One participant from the control group completed the resting state scan but not the CTQ or the TAS-20 and was subsequently removed from analyses involving either questionnaire. We focused on the FI-DMN, DMN, and DMN-OSM to investigate the associations based on our a priori hypothesis, with an 2-sided significance level of *P* = .05. Post hoc Pearson correlations determined the direction of the association in each group.

#### Moderated Mediation

Moderated mediation was used to assess whether there was a group difference in the association between total CTQ score, total alexithymia score, and time spent in the FI-DMN state using the mediation package, version 4.5.0, in R, version 4.3.0. We tested the significance of this indirect effect using bootstrapping procedures. Unstandardized indirect effects were computed for each of 1000 bootstrapped samples, and the 95% CI was computed by calculating the indirect effects at the 2.5th and 97.5th percentiles. To assess the indirect effects for each group, we completed 2 separate follow-up mediations.

## Results

### Group Differences in Dynamic Brain States

**Figure 2.**
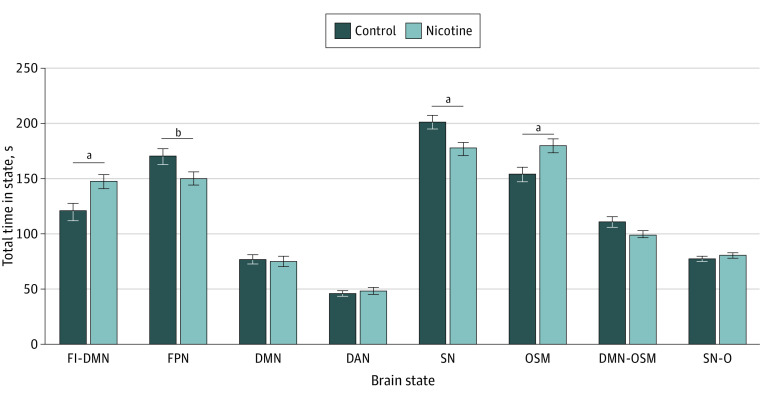
Total Time in Brain State Error bars represent SEs. DAN indicates dorsal attention network; DMN, default mode network; FI, frontoinsular; FPN, frontoparietal network; OSM, occipital sensory-motor; and SN, salience network. ^a^*P* < .05. ^b^*P* < .01.

This analysis included 204 participants (mean [SD] age, 37.53 [10.64] years; 95 [46.6%] female; 109 [53.4%] male), including 102 individuals who smoked nicotine long term and 102 healthy controls who did not smoke. A total of 77 (37.7%) were African American or Black; 11 (5.4%), Asian; 8 (3.9%) Hispanic; 194 (95.1%), non-Hispanic; 104 (51.0%), White; 12 (5.9%), more than 1 race; and 2 (1.0%), unknown or unreported ethnicity. The 3T Trio MRI scanner was used for 159 participants (77.9%) and the Prisma scanner for 45 (22.1%). Repeated-measures ANOVA on time spent in state revealed a significant group × state interaction (η^2^*_p_* = 0.12; *P* = .001) and a significant main effect of state (η^2^*_p_* = 0.68; *P* < .001), controlling for scanner type. Due to the standardized scan time, there was no main effect of group on total time in all states. A Greenhouse-Geisser correction value of 0.26 was used. In post hoc tests to evaluate the interaction of group and state, compared with controls, individuals who smoked nicotine spent significantly more total time in the FI-DMN (mean difference, 25.63 seconds; 95% CI, 8.05-43.20 seconds; η^2^*_p_* = 0.04; *P* = .004 after correction) and the OSM (mean difference, 23.60 seconds; 95% CI, 6.36-40.85 seconds; η^2^*_p_* = 0.04; *P* = .008 after correction) states (Figure 2) and less total time in the SN (mean difference, 23.47 seconds; 95% CI, 9.66-37.28 seconds; η^2^*_p_* = 0.05; *P* < .001 after correction) and frontoparietal network (FPN) (mean difference, 19.63 seconds; 95% CI, 2.51-36.75 seconds; η^2^*_p_* = 0.03; *P* = .03 after correction) states.

Repeated-measures ANOVA on persistence in state revealed a significant main effect of state (η^2^*_p_* = 0.28; *P* < .001) and a significant group × state interaction (η^2^*_p_* = 0.08; *P* = .03), controlling for scanner type. A Greenhouse-Geisser correction factor of 0.48 was used. In a post hoc test, individuals who smoked nicotine exhibited significantly shorter persistence in the FPN (η^2^*_p_* = 0.02; *P* = .03 after correction) and SN (η^2^*_p_* = 0.04; *P* = .006 after correction) states compared with controls (eFigure 2 in Supplement 1).

The total number of transitions to each state in each group was 9087 among controls and 8710 among individuals who smoked. Two-proportion *z*-tests indicated that individuals who smoked nicotine transitioned significantly more to FI-DMN (difference in proportion, 0.02; 95% CI, 0.01-0.03; *P* < .001 after correction) and OSM (difference in proportion, 0.02; 95% CI, 0.01-0.03; *P* < .001 after correction) states while transitioning significantly less to FPN (difference in proportion, −0.02; 95% CI, −0.03 to −0.007; *P* = .01 after correction) and SN (difference in proportion, −0.03; 95% CI, −0.04 to −0.02; *P* < .001 after correction) states compared with the control group (eFigure 2 in Supplement 1).

### Correlations of Brain States With Alexithymia and Childhood Trauma

**Figure 3.**
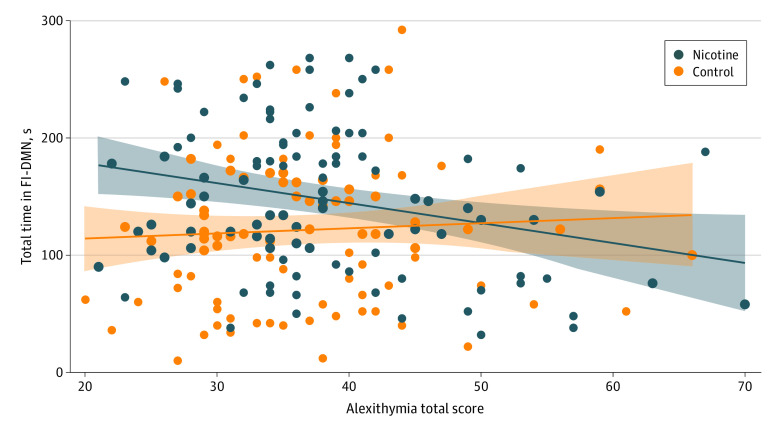
Correlation Between Time in Frontoinsular-Default Mode Network (FI-DMN) State and Alexithymia as a Function of Group For individuals who used nicotine long term, greater alexithymia was associated with less time in the FI-DMN state. For healthy control individuals, there was no association. Data markers indicate all participant data; diagonal lines, associations between alexithymia and time in the FI-DMN state; and shading, SEs.

In a linear model including alexithymia, group, and state and their interaction, covarying for scanner type, we found a 3-way interaction of group × state × alexithymia (η^2^*_p_* = 0.001; *P* = .03), a significant group × alexithymia interaction (η^2^*_p_* = 0.0001; *P* = .01), and a state × alexithymia interaction (η^2^*_p_* = 0.01; *P* = .03). Post hoc analyses focused on the group × alexithymia interaction in the DMN states. We found an alexithymia × group interaction (η^2^*_p_* = 0.02; *P* = .04) in the FI-DMN state. Among individuals who smoked, time in the FI-DMN state was negatively correlated with their alexithymia total score (*r*, −0.26; 95% CI, −0.44 to −0.07; *P* = .007), whereas in the control group, there was no correlation (*r*, 0.06; 95% CI, −0.14 to 0.25; *P* = .54) (Figure 3). No associations with alexithymia were observed for the DMN or DMN-OSM states.

The linear regression found no significant interaction of CTQ × state or CTQ × state × group. In a linear model, we found a significant main effect of CTQ, but no interaction of CTQ × group or main effect of group. Higher CTQ scores were associated with higher alexithymia scores (*r*, 0.21; 95% CI, 0.08-0.34; *P* = .002). Of note, mean (SD) CTQ scores for controls (35.67 [13.69]) and individuals who smoked (41.94 [17.05]) significantly differed (*P* = .005); however the mean (SD) alexithymia scores did not (controls: 38.22 [9.72]; nicotine group: 36.65 [8.59]; *P* = .24).

### Mediation Analyses

In moderated mediation analysis, the independent variable was the total CTQ score, the alexithymia score was the mediator, total time in the FI-DMN state was the dependent variable, and group was the moderator variable. The difference between the 2 indirect effects was significant (*c*′ = 0.28; *P* = .02), but the difference between the direct effects was not significant (*c* = −0.39; *P* = .41).

**Figure 4.**
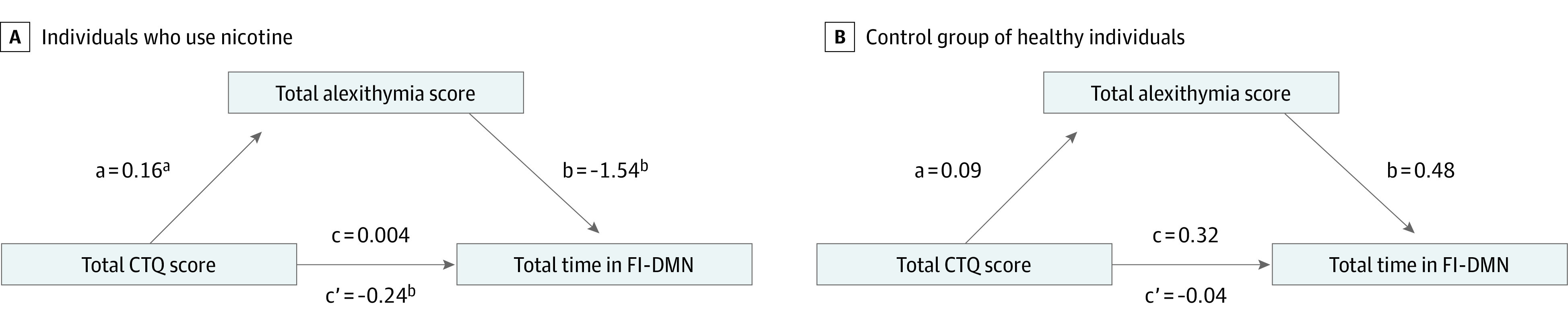
Mediation of the Associations Between Childhood Trauma, Alexithymia, and Time in the Frontoinsular Default Mode Network (FI-DMN) State (A) Unstandardized regression coefficients for the association between childhood trauma and time in the FI-DMN state, as mediated by alexithymia in individuals who smoked nicotine long term. There was a significant association between childhood trauma and alexithymia and alexithymia and time in the FI-DMN state. The indirect mediated pathway was significant. (B) Unstandardized regression coefficients for the association between childhood trauma and time in the FI-DMN state, as mediated by alexithymia in healthy controls. There was no direct or indirect association in this model. CTQ indicates Childhood Trauma Questionnaire. ^a^*P* < .05. ^b^*P* < .01.

To determine the direction of the association in each group, we completed 2 separate mediations. For individuals who smoked nicotine, the association of the total childhood trauma score with total time in the FI-DMN state was fully mediated via their total alexithymia score (Figure 4A) (bootstrapped unstandardized indirect effect [c′], −0.24; 95% CI, −0.58 to −0.03; *P* = .02). For controls, the association of the total childhood trauma score with total time in the FI-DMN was not mediated by the total alexithymia score (unstandardized indirect effect [c′], 0.04; 95% CI, −0.11 to 0.28; *P* = .59) (Figure 4B).

## Discussion

The current study defined differences in brain dynamics as a function of nicotine use and found an association of temporal dynamics with childhood trauma–related alexithymia in those who smoked nicotine. When only considering smoking status, those who smoked spent significantly more time in the FI-DMN and OSM states and less time in the SN and FPN states compared with those who did not smoke. The group difference in total time spent in these states was explained by group differences in the number of transitions made into each state. The OSM finding is consistent with literature showing that nicotine enhances sensorimotor function,^38,39^ while group differences in the other networks are consistent with prior work showing that substance use is associated with executive control deficits,^40^ greater engagement of the DMN,^13^ and more integration between the DMN and SN.^12,13,41^ Furthermore, FI-DMN network engagement is associated with rumination^7^ and affective aspects of self-referential processing,^42^ which may in turn contribute to craving and substance use. This is because the FI-DMN represents the coordinated activity of both the DMN and the SN regions, which are typically discussed as separate networks yet become more functionally connected during nicotine craving and withdrawal.^12,13,41^ Thus, the present study offers novel insight into the temporal engagement of coordinated activity of 2 networks that often work together to maintain nicotine dependence.

Our current work also revealed an association of childhood trauma, mediated through alexithymia, with FI-DMN function in those who smoked nicotine. Specifically, more reported childhood trauma was associated with greater alexithymia, replicating previous work.^26^ However, no direct association was evident between the CTQ score and time in any brain state. Only through the association of childhood trauma with alexithymia did we find an association with neurobiological changes in individuals who smoked nicotine, indicating that it was the expression of symptoms such as alexithymia and not just trauma that was associated with FI-DMN dynamics. This is consistent with prior work showing that alexithymia mediates the association between childhood trauma and adult psychiatric symptoms.^27^ The fact that an association between trauma-related alexithymia and temporal dynamics was found only in those who smoked is consistent with findings showing an association between alexithymia and reduced connectivity between regions of the DMN and SN in those with substance use disorder but not in controls.^12,24^ Interestingly, while more time in the FI-DMN state was observed in those who smoked nicotine compared with controls, trauma-related alexithymia was associated with less time spent in this same network state in those who smoked nicotine. This is consistent with previous literature indicating that there are neurobiological differences within psychiatric diagnoses depending on whether individuals have been maltreated or not^43^ and suggests that specific neurobiological risk profiles are associated with trauma-related factors. Together with the current findings, we suggest that too much or too little FI-DMN function may correspond with different aspects of emotional dysregulation (ie, rumination and alexithymia, respectively), both of which contribute to nicotine craving and use.^25^ Our findings further suggest that there are multiple neurobiological mechanisms of substance use, and some are associated with trauma-related alexithymia.

### Limitations

While the current study provides a novel understanding of the neural correlates of nicotine use and its connection with alexithymia and childhood trauma, several limitations and future directions should be acknowledged. First, as the current study only measured brain dynamics following ad lib smoking, we are unable to comment on the direct pharmacological influence of nicotine. We were also unable to determine whether the propensity to spend more time in the FI-DMN state was a preexisting risk factor for nicotine use or the consequence of long-term nicotine use. Finally, it is unclear whether the lack of correlation between alexithymia and FI-DMN engagement was due to a learned maladaptive emotional coping style or to trauma-induced changes in neurobiological function, as the FI-DMN state includes brain regions impacted by childhood maltreatment.^44,45^ Nevertheless, the present findings provide critical data for future longitudinal work, especially in populations with psychopathology or with a broader range of severity of childhood trauma and/or alexithymia, and may provide a more nuanced understanding of the interaction between childhood trauma, alexithymia, and neurobiology.

## Conclusions

The results of this cross-sectional study demonstrated not only differences in temporal dynamics between those who do and do not smoke nicotine but also an association between childhood trauma, alexithymia, and brain dynamics in individuals with nicotine dependence. This work showed that, on average, individuals who smoked nicotine spent more time in the FI-DMN state, which may increase rumination and craving.^7,42^ However, this study showed an association between trauma-related alexithymia and nicotine use, suggesting that for some individuals with childhood maltreatment, smoking may be driven by blunted affective awareness related to diminished FI-DMN engagement. The findings therefore point to the need to evaluate the impact of developmental trauma and transdiagnostic factors such as alexithymia when considering links between neurobiological profiles and substance use, as doing so may reveal meaningful neurobiological variance that is otherwise obscured when only considering group differences.

## Notes

Supplement 1. **eFigure 1.** Coactivation Patterns Visualized in MNI Space—Full Slice Sequence **eFigure 2.** Persistence in and Transitions to Brain States **eAppendix.** fMRI Preprocessing and Analysis
eReferences

Supplement 2.
Data Sharing Statement
