# Supplementary material for: Childhood Trauma, Emotional Awareness, and Neural Correlates of Long-Term Nicotine Smoking
Source: JAMA Netw Open. 2024 Jan 11;7(1):e2351132. doi: 10.1001/jamanetworkopen.2023.51132 (PMC10784870; doi:10.1001/jamanetworkopen.2023.51132)
Supplement: Supplement 1. — eFigure 1. Coactivation Patterns Visualized in MNI Space—Full Slice Sequence eFigure 2. Persistence in and Transitions to Brain States eAppendix. fMRI Preprocessing and Analysis eReferences [file jamanetwopen-e2351132-s001.pdf]

# Supplementary Online Content

Quam A, Biernacki K, Ross TJ, Salmeron BJ, Janes AC. Childhood trauma, emotional awareness, and neural correlates of long-term nicotine smoking. *JAMA Netw Open*. 2024;7(1):e2351132. doi:10.1001/jamanetworkopen.2023.51132

**eFigure 1.** Coactivation Patterns Visualized in MNI Space—Full Slice Sequence
**eFigure 2.** Persistence in and Transitions to Brain States
**eAppendix.** fMRI Preprocessing and Analysis
**eReferences**

This supplementary material has been provided by the authors to give readers additional information about their work.

© 2024 Quam A et al. *JAMA Network Open*.

**eFigure 1. Coactivation Patterns visualized in MNI space – full slice sequence. All are in neurologic convention (left brain on the left).**

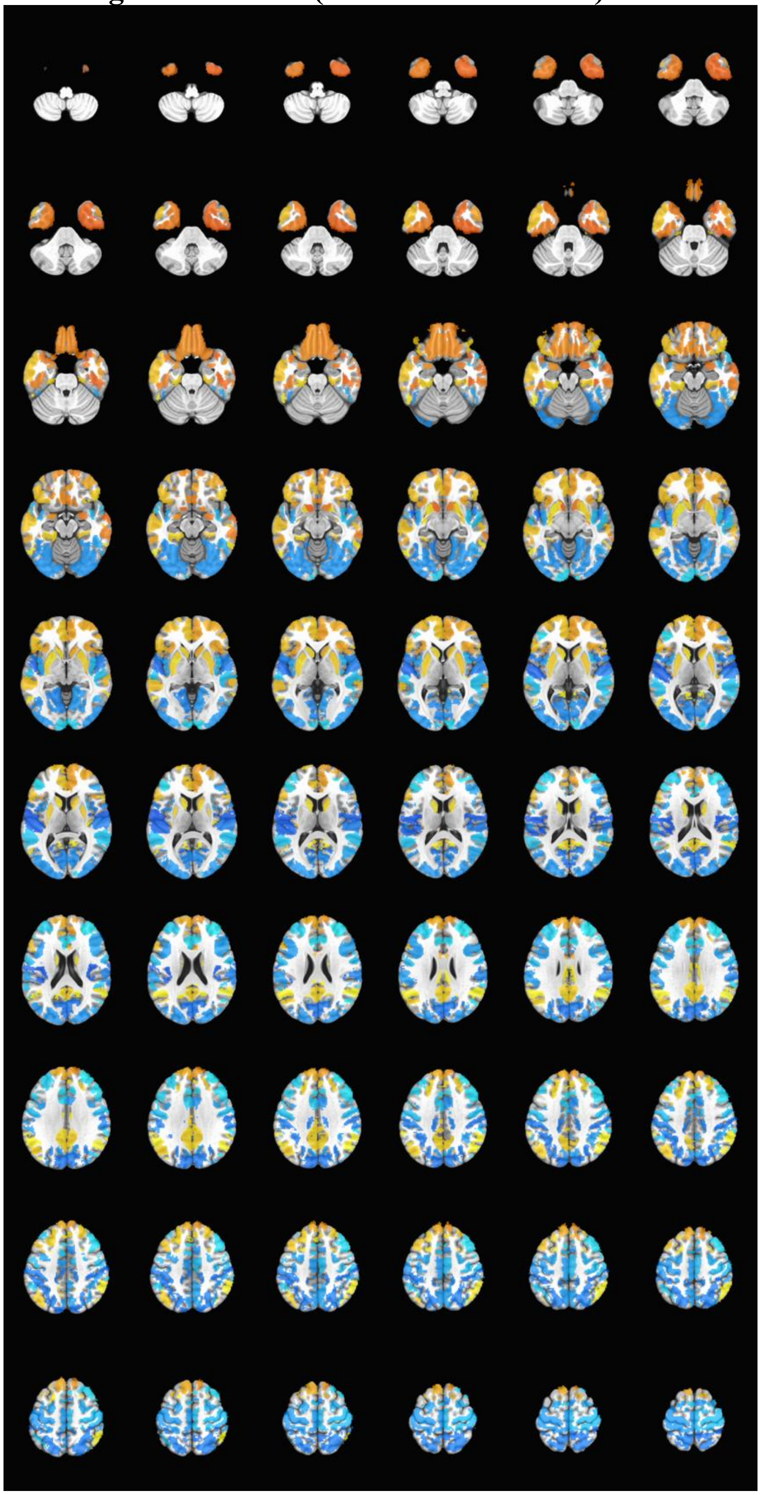

***eFigure 1A.*** *Frontoinsular default mode network (FI-DMN).*

© 2024 Quam A et al. *JAMA Network Open*.

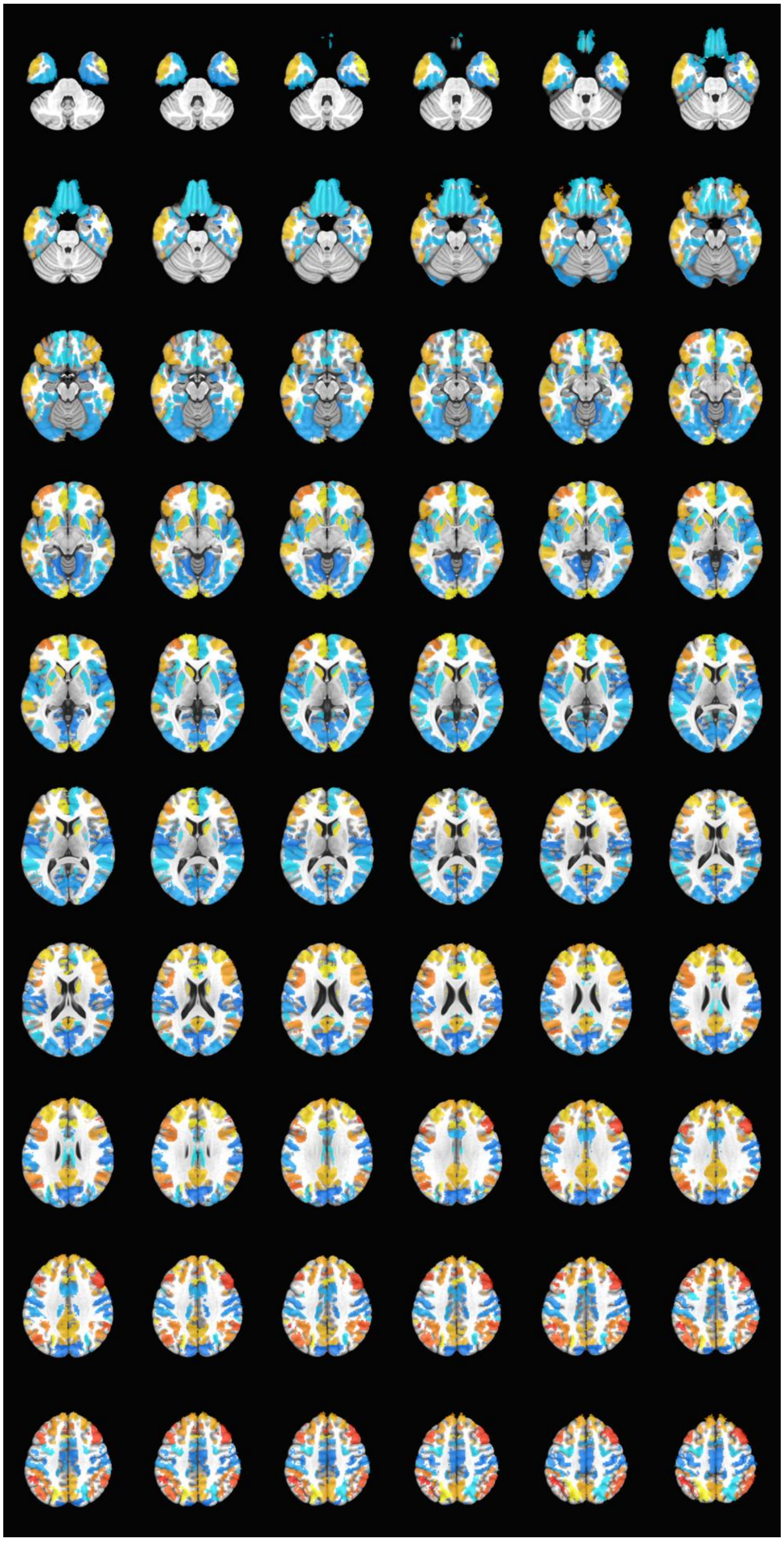

***eFigure 1B.*** *Frontoparietal network (FPN).*

© 2024 Quam A et al. *JAMA Network Open*.

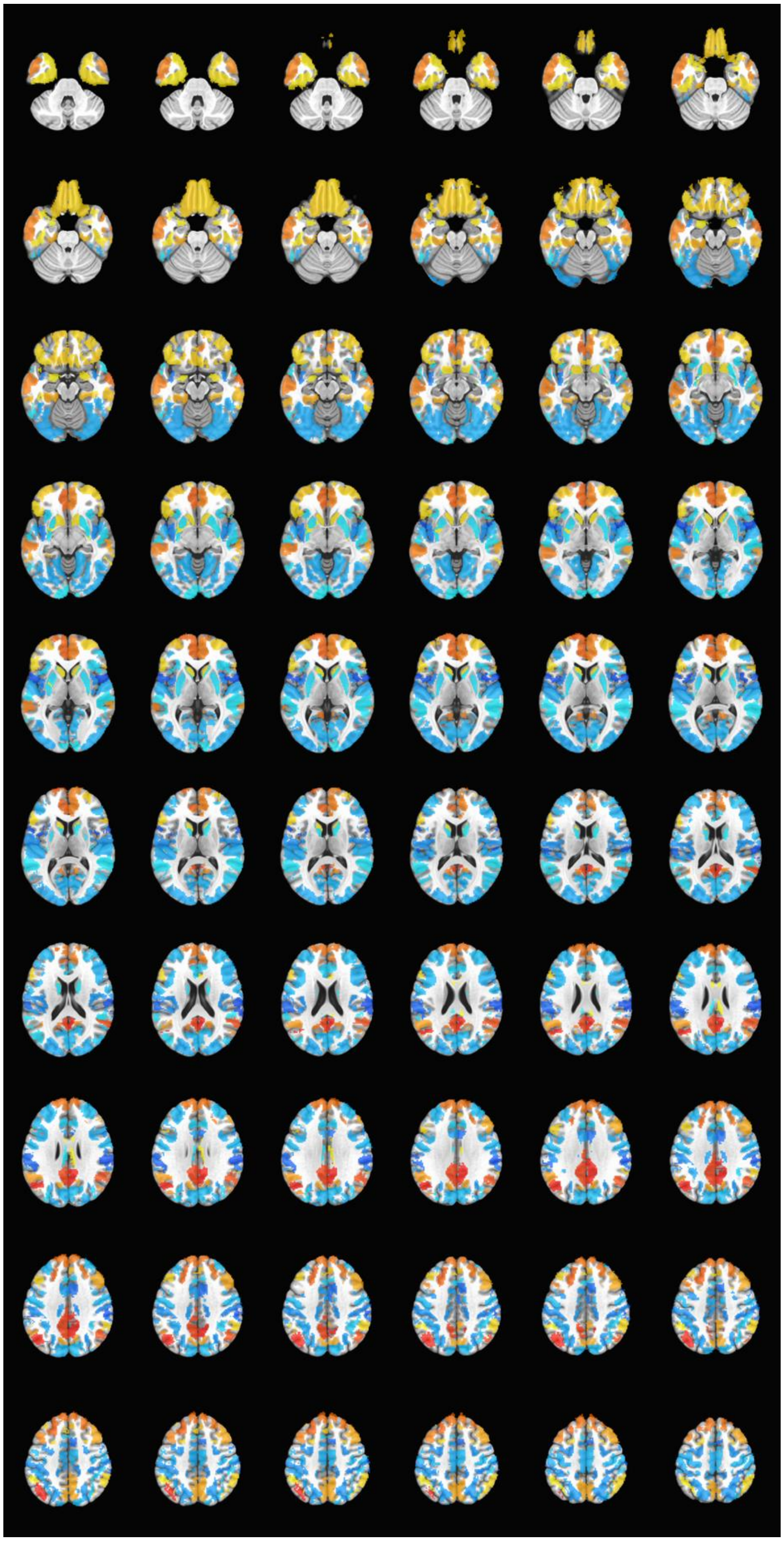

***eFigure 1C.*** *Default mode network (DMN).*

© 2024 Quam A et al. *JAMA Network Open*.

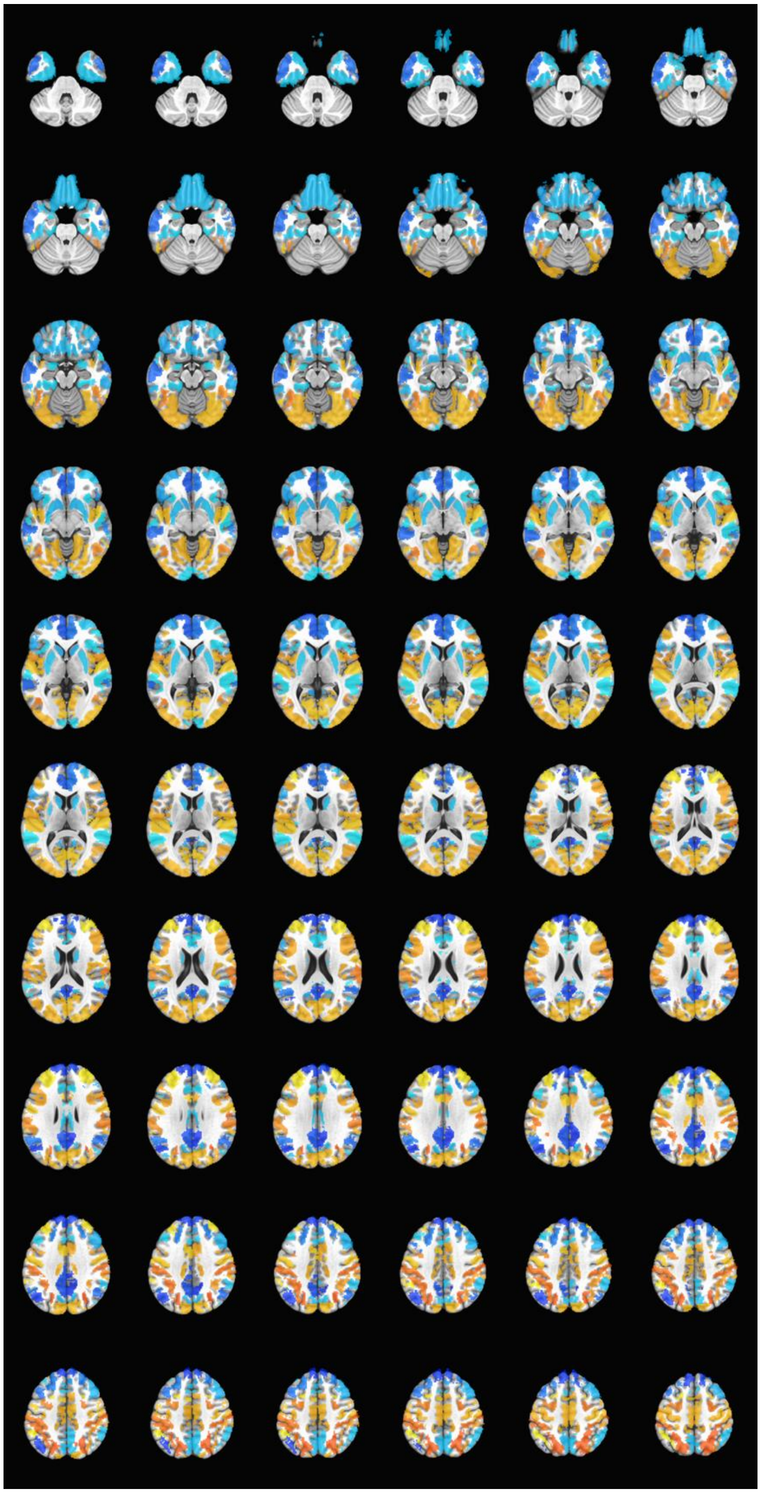

***eFigure 1D.*** *Dorsal attention network (DAN).*

© 2024 Quam A et al. *JAMA Network Open*.

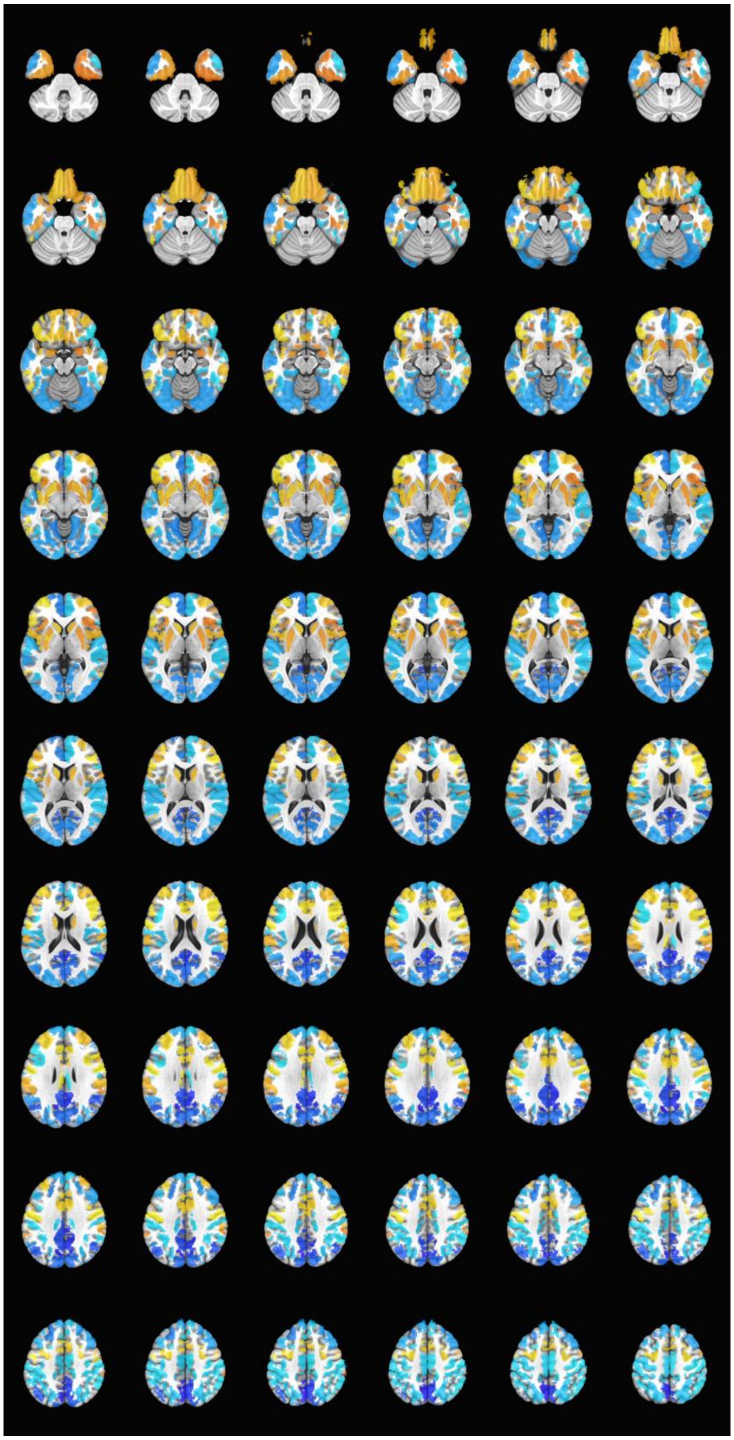
***eFigure 1E.*** *Salience network (SN).*

© 2024 Quam A et al. *JAMA Network Open*.

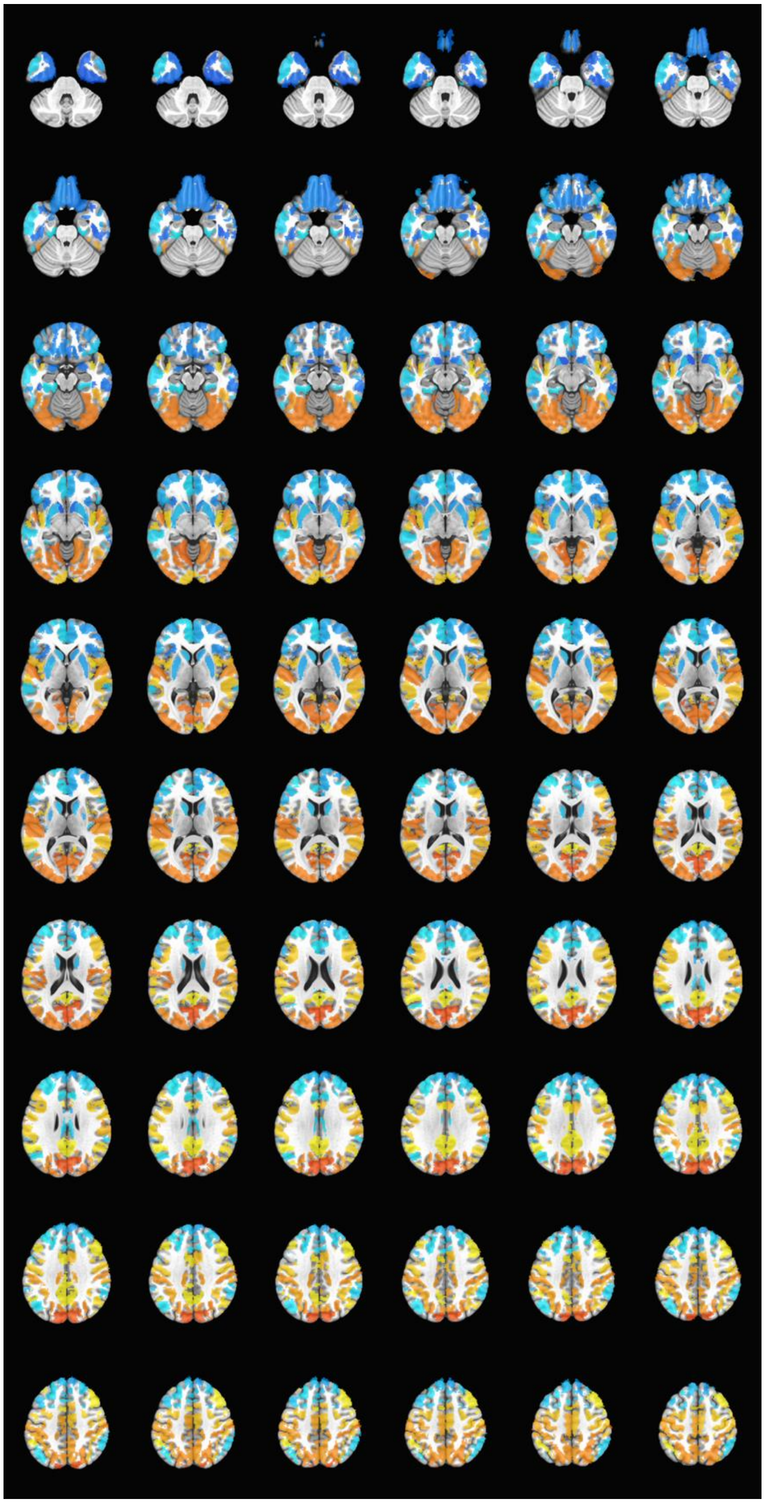

***eFigure 1F.*** *Occipital sensorimotor network (OSM).*

© 2024 Quam A et al. *JAMA Network Open*.

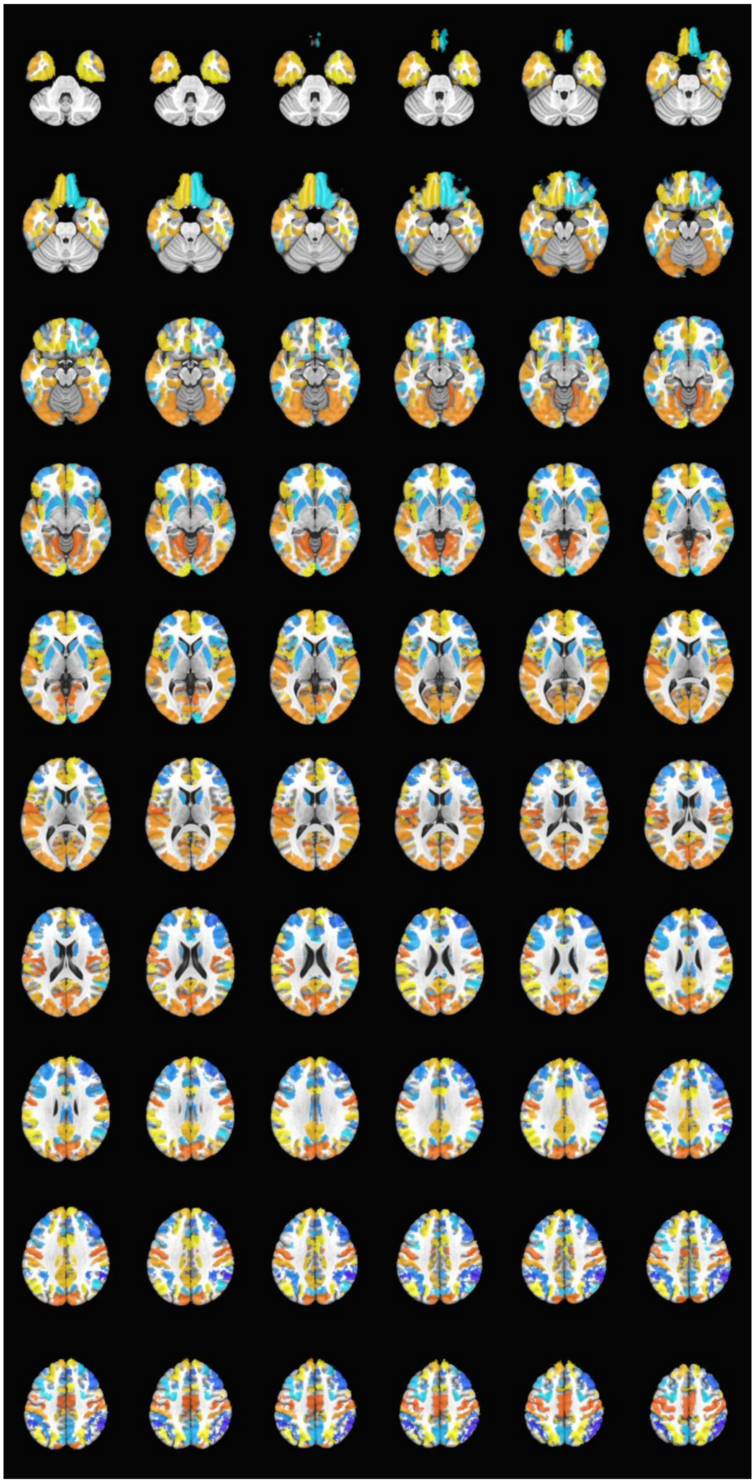

***eFigure 1G.*** *Default mode network with Occipital cortex (DMN-OSM).*

© 2024 Quam A et al. *JAMA Network Open*.

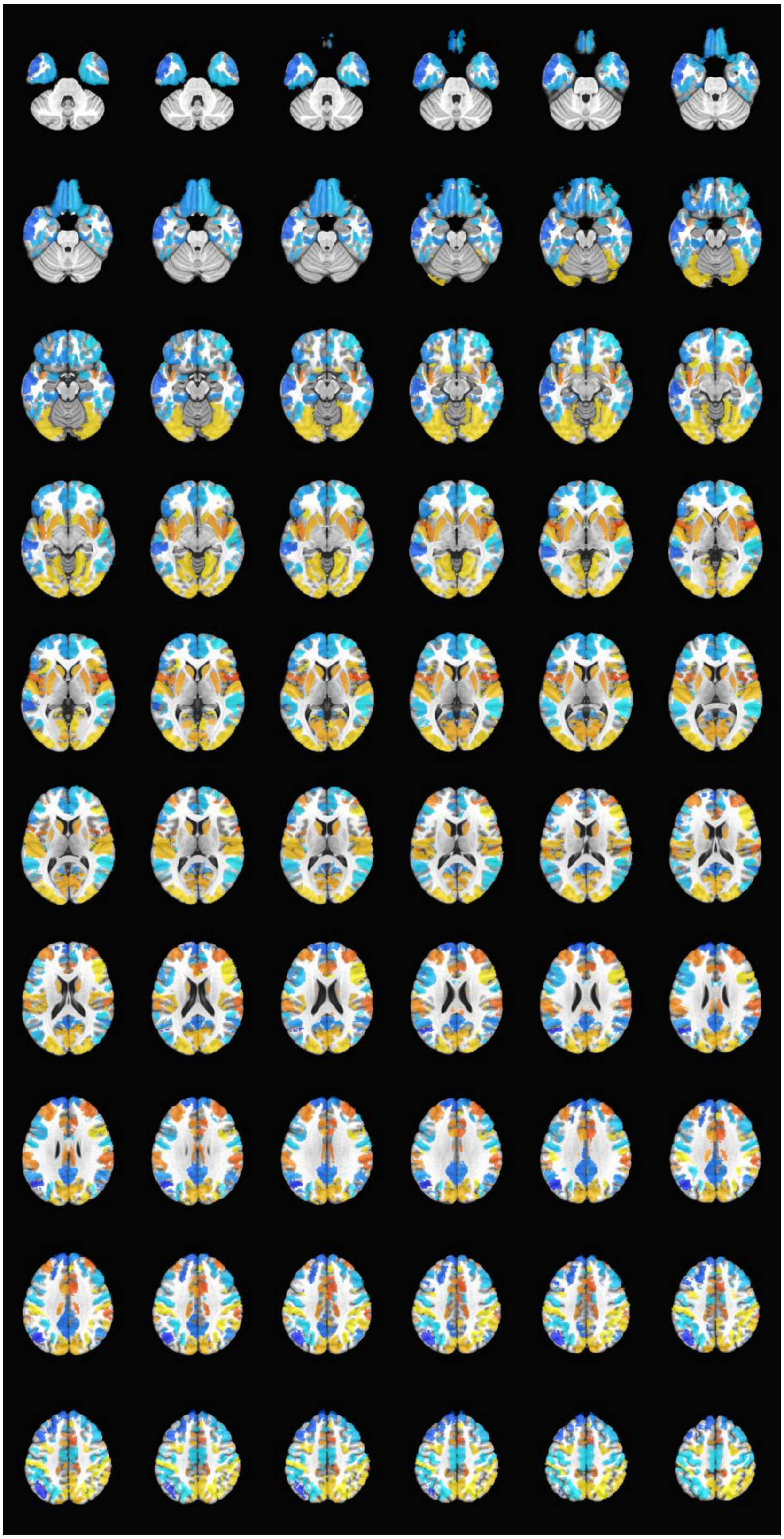

***eFigure 1H.*** *Salience and Occipital network (SN-O).*

© 2024 Quam A et al. *JAMA Network Open*.

**eFigure 2. Persistence in and Transitions to Brain States**

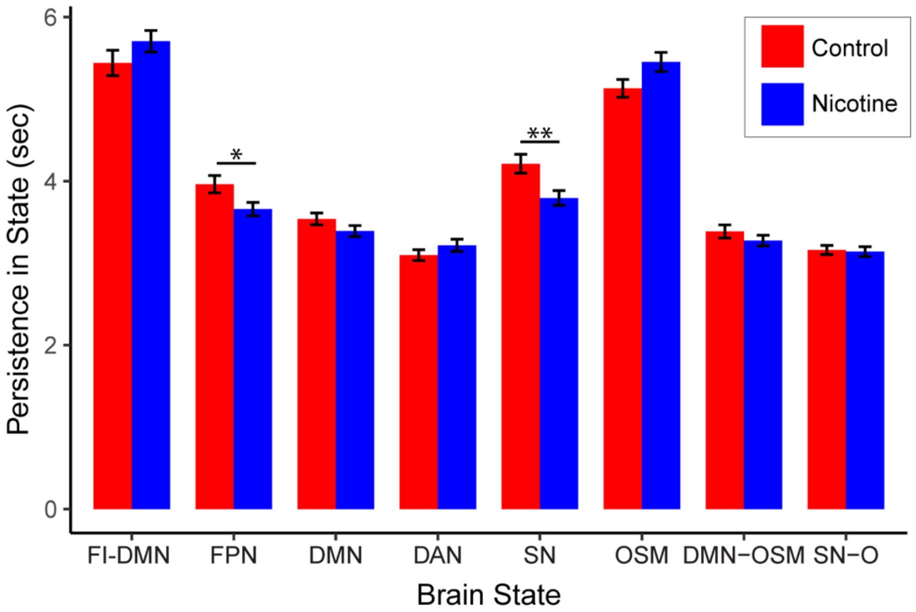


***eFigure 2A.*** *Persistence in brain state.* The average persistence in each brain state by group. Controls persistent in states FPN and SN-1 significantly more than nicotine users. Error bars represent standard error. *: $p < 0.05$, **: $p < 0.01$

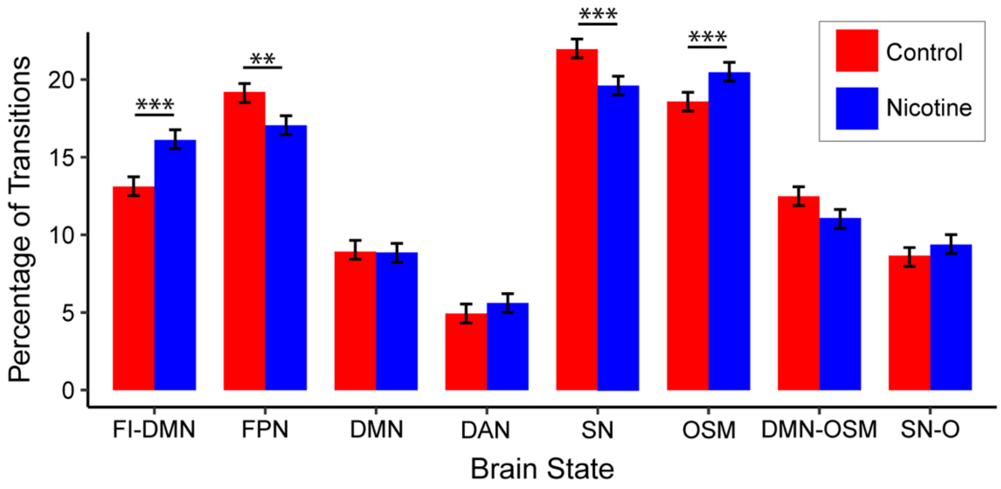


***eFigure 2B.*** *Transitions to brain states.* Two-proportion z-tests indicate nicotine users transitioned more frequently than controls into the DMN-FI and OSM states, while transitioning less into the FPN and SN-1 states. Error bars represent standard error. ** $p = 0.01$; *** $p < 0.001$

© 2024 Quam A et al. *JAMA Network Open*.

**eAppendix. fMRI preprocessing and analysis**

The scan parameters for the T2-weighted images to detect the blood oxygenation level-dependent (BOLD) effects include: repetition time (TR) = 2000 ms, echo time (TE) = 27.0 ms, flip angle= 78 degrees, slice thickness = 4 mm, voxel size = 3.4375 x 3.4375 x 4 $mm^3$ number of slices = 39. T1-weighted anatomical images were acquired using the following parameters: TR = 1900 ms, TE = 3.51 ms, flip angle = 9 degrees, slice thickness = 1 mm, voxel size= 1.0 x 1.0 x 1.0 $mm^3$, number of slices = 208

Neuroimaging data was preprocessed using fmriprep version 20.2.7. The following description of the methods was generated and copied from their website:

*Results included in this manuscript come from preprocessing performed using FMRIPREP version 20.2.7 [1, 2, RRID:SCR_016216], a Nipype [3, 4, RRID:SCR_002502] based tool. Each T1w (T1-weighted) volume was corrected for INU (intensity non-uniformity) using `N4BiasFieldCorrection` v2.1.0 [5] and skull-stripped using `antsBrainExtraction.sh` v2.1.0 (using the OASIS template). Brain surfaces were reconstructed using `recon-all` from FreeSurfer v6.0.1 [6, RRID:SCR_001847], and the brain mask estimated previously was refined with a custom variation of the method to reconcile ANTs-derived and FreeSurfer-derived segmentations of the cortical gray-matter of Mindboggle [21, RRID:SCR_002438]. Spatial normalization to the ICBM 152 Nonlinear Asymmetrical template version 2009c [7, RRID:SCR_008796] was performed through nonlinear registration with the `antsRegistration` tool of ANTs v2.1.0 [8, RRID:SCR_004757], using brain-extracted versions of both T1w volume and template. Brain tissue segmentation of cerebrospinal fluid (CSF), white-matter (WM) and gray-matter (GM) was performed on the brain-extracted T1w using `fast` [17] (FSL v5.0.9, RRID:SCR_002823).*

*Functional data was slice time corrected using `3dTshift` from AFNI v16.2.07 [11, RRID:SCR_005927] and motion corrected using `mcflirt` (FSL v5.0.9 [9]). This was followed by co-registration to the corresponding T1w using boundary-based registration [16] with six degrees of freedom, using `bbregister` (FreeSurfer v6.0.1). Motion correcting transformations, BOLD-to-T1w transformation and T1w-to-template (MNI) warp were concatenated and applied in a single step using `antsApplyTransforms` (ANTs v2.1.0) using Lanczos interpolation.*

*Physiological noise regressors were extracted applying CompCor [18]. Principal components were estimated for the two CompCor variants: temporal (tCompCor) and anatomical*

© 2024 Quam A et al. *JAMA Network Open*.

*(aCompCor). A mask to exclude signal with cortical origin was obtained by eroding the brain mask, ensuring it only contained subcortical structures. Six tCompCor components were then calculated including only the top 5% variable voxels within that subcortical mask. For aCompCor, six components were calculated within the intersection of the subcortical mask and the union of CSF and WM masks calculated in T1w space, after their projection to the native space of each functional run. Frame-wise displacement [19] was calculated for each functional run using the implementation of Nipype. ICA-based Automatic Removal Of Motion Artifacts (AROMA) was used to generate aggressive noise regressors as well as to create a variant of data that is non-aggressively denoised [20].*

*Many internal operations of FMRIPREP use Nilearn [22, RRID:SCR_001362], principally within the BOLD-processing workflow. For more details of the pipeline see [https://fmriprep.readthedocs.io/en/20.2.7/workflows.html](https://fmriprep.readthedocs.io/en/20.2.7/workflows.html).*

© 2024 Quam A et al. *JAMA Network Open*.

**eReferences**


1. Esteban O, Markiewicz CJ, Blair RW, Moodie CA, Isik AI, Erramuzpe A, Kent JD, Goncalves M, DuPre E, Snyder M, Oya H, Ghosh SS, Wright J, Durnez J, Poldrack RA, Gorgolewski KJ. fMRIPrep: a robust preprocessing pipeline for functional MRI. Nat Meth. 2018; doi:10.1038/s41592-018-0235-4

2. fMRIPrep Available from: 10.5281/zenodo.852659.

3. Gorgolewski K, Burns CD, Madison C, Clark D, Halchenko YO, Waskom ML, Ghosh SS. Nipype: a flexible, lightweight and extensible neuroimaging data processing framework in python. Front Neuroinform. 2011 Aug 22;5(August):13. doi:10.3389/fninf.2011.00013.

4. Gorgolewski KJ, Esteban O, Ellis DG, Notter MP, Ziegler E, Johnson H, Hamalainen C, Yvernault B, Burns C, Manhães-Savio A, Jarecka D, Markiewicz CJ, Salo T, Clark D, Waskom M, Wong J, Modat M, Dewey BE, Clark MG, Dayan M, Loney F, Madison C, Gramfort A, Keshavan A, Berleant S, Pinsard B, Goncalves M, Clark D, Cipollini B, Varoquaux G, Wassermann D, Rokem A, Halchenko YO, Forbes J, Moloney B, Malone IB, Hanke M, Mordom D, Buchanan C, Pauli WM, Huntenburg JM, Horea C, Schwartz Y, Tungaraza R, Iqbal S, Kleesiek J, Sikka S, Frohlich C, Kent J, Perez-Guevara M, Watanabe A, Welch D, Cumba C, Ginsburg D, Eshaghi A, Kastman E, Bougacha S, Blair R, Acland B, Gillman A, Schaefer A, Nichols BN, Giavasis S, Erickson D, Correa C, Ghayoor A, Küttner R, Haselgrove C, Zhou D, Craddock RC, Haehn D, Lampe L, Millman J, Lai J, Renfro M, Liu S, Stadler J, Glatard T, Kahn AE, Kong X-Z, Triplett W, Park A, McDermottroe C, Hallquist M, Poldrack R, Perkins LN, Noel M, Gerhard S, Salvatore J, Mertz F, Broderick W, Inati S, Hinds O, Brett M, Durnez J, Tambini A, Rothmei S, Andberg SK, Cooper G, Marina A, Mattfeld A, Urchs S, Sharp P, Matsubara K, Geisler D, Cheung B, Floren A, Nickson T, Pannetier N, Weinstein A, Dubois M, Arias J, Tarbert C, Schlamp K, Jordan K, Liem F, Saase V, Harms R, Khanuja R, Podranski K, Flandin G, Papadopoulos Orfanos D, Schwabacher I, McNamee D, Falkiewicz M, Pellman J, Linkersdörfer J, Varada J, Pérez-García F, Davison A, Shachnev D, Ghosh S. Nipype: a flexible, lightweight and extensible neuroimaging data processing framework in Python. 2017. doi:10.5281/zenodo.581704.


© 2024 Quam A et al. *JAMA Network Open*.

5. Tustison NJ, Avants BB, Cook PA, Zheng Y, Egan A, Yushkevich PA, Gee JC. N4ITK: improved N3 bias correction. IEEE Trans Med Imaging. 2010 Jun;29(6):1310–20. doi:10.1109/TMI.2010.2046908.

6. Dale A, Fischl B, Sereno MI. Cortical Surface-Based Analysis: I. Segmentation and Surface Reconstruction. Neuroimage. 1999;9(2):179–94. doi:10.1006/nimg.1998.0395.

7. Fonov VS, Evans AC, McKinstry RC, Almli CR, Collins DL. Unbiased nonlinear average age-appropriate brain templates from birth to adulthood. NeuroImage; Amsterdam. 2009 Jul 1;47:S102. doi:10.1016/S1053-8119(09)70884-5.

8. Avants BB, Epstein CL, Grossman M, Gee JC. Symmetric diffeomorphic image registration with cross-correlation: evaluating automated labeling of elderly and neurodegenerative brain. Med Image Anal. 2008 Feb;12(1):26–41. doi:10.1016/j.media.2007.06.004.

9. Jenkinson M, Bannister P, Brady M, Smith S. Improved optimization for the robust and accurate linear registration and motion correction of brain images. Neuroimage. 2002 Oct;17(2):825–41. doi:10.1006/nimg.2002.1132.

10. Andersson JLR, Skare S, Ashburner J. How to correct susceptibility distortions in spin-echo echo-planar images: application to diffusion tensor imaging. Neuroimage. 2003 Oct;20(2):870–88. doi:10.1016/S1053-8119(03)00336-7.

11. Cox RW. AFNI: software for analysis and visualization of functional magnetic resonance neuroimages. Comput Biomed Res. 1996 Jun;29(3):162–73. doi:10.1006/cbmr.1996.0014.

12. Jenkinson M. Fast, automated, N-dimensional phase-unwrapping algorithm. Magn Reson Med. 2003 Jan;49(1):193–7. doi:10.1002/mrm.10354.

13. Huntenburg JM. Evaluating nonlinear coregistration of BOLD EPI and T1w images. Freie Universität Berlin; 2014. Available from: http://hdl.handle.net/11858/00-001M-0000-002B-1CB5-A.

14. Wang S, Peterson DJ, Gatenby JC, Li W, Grabowski TJ, Madhyastha TM. Evaluation of Field Map and Nonlinear Registration Methods for Correction of Susceptibility Artifacts in Diffusion MRI. Front Neuroinform. 2017 [cited 2017 Feb 21];11. doi:10.3389/fninf.2017.00017.

© 2024 Quam A et al. *JAMA Network Open*.

15. Treiber JM, White NS, Steed TC, Bartsch H, Holland D, Farid N, McDonald CR, Carter BS, Dale AM, Chen CC. Characterization and Correction of Geometric Distortions in 814 Diffusion Weighted Images. PLoS One. 2016 Mar 30;11(3):e0152472. doi:10.1371/journal.pone.0152472.

16. Greve DN, Fischl B. Accurate and robust brain image alignment using boundary-based registration. Neuroimage. 2009 Oct;48(1):63–72. doi:10.1016/j.neuroimage.2009.06.060.

17. Zhang Y, Brady M, Smith S. Segmentation of brain MR images through a hidden Markov random field model and the expectation-maximization algorithm. IEEE Trans Med Imaging. 2001 Jan;20(1):45–57. doi:10.1109/42.906424.

18. Behzadi Y, Restom K, Liau J, Liu TT. A component based noise correction method (CompCor) for BOLD and perfusion based fMRI. Neuroimage. 2007 Aug 1;37(1):90–101. doi:10.1016/j.neuroimage.2007.04.042.

19. Power JD, Mitra A, Laumann TO, Snyder AZ, Schlaggar BL, Petersen SE. Methods to detect, characterize, and remove motion artifact in resting state fMRI. Neuroimage. 2013 Aug 29;84:320–41. doi:10.1016/j.neuroimage.2013.08.048.

20. Pruim RHR, Mennes M, van Rooij D, Llera A, Buitelaar JK, Beckmann CF. ICA-AROMA: A robust ICA-based strategy for removing motion artifacts from fMRI data. Neuroimage. 2015 May 15;112:267–77. doi:10.1016/j.neuroimage.2015.02.064.

21. Klein A, Ghosh SS, Bao FS, Giard J, Häme Y, Stavsky E, et al. Mindboggling morphometry of human brains. PLoS Comput Biol 13(2): e1005350. 2017. doi:10.1371/journal.pcbi.1005350.

22. Abraham A, Pedregosa F, Eickenberg M, Gervais P, Mueller A, Kossaifi J, Gramfort A, Thirion B, Varoquaux G. Machine learning for neuroimaging with scikit-learn. Front in Neuroinf 8:14. 2014. doi:10.3389/fninf.2014.00014.

© 2024 Quam A et al. *JAMA Network Open*.
