# Supplementary material for: Childhood Trauma, Emotional Awareness, and Neural Correlates of Long-Term Nicotine Smoking
Source: JAMA Netw Open. 2024 Jan 11;7(1):e2351132. doi: 10.1001/jamanetworkopen.2023.51132 (PMC10784870; doi:10.1001/jamanetworkopen.2023.51132)
Supplement: Supplement 2. — Data Sharing Statement [file jamanetwopen-e2351132-s002.pdf]

Data Sharing Statement

Quam. Childhood Trauma, Emotional Awareness, and Neural Correlates of Long-Term Nicotine Smoking. *JAMA Netw Open*. Published January 11, 2024.
doi:10.1001/jamanetworkopen.2023.51132

Data
Data available: Yes
Data types: Deidentified participant data
How to access data: Data may be requested by emailing amy.janes@nih.gov.
When available: With publication

Supporting Documents
Document types: Other (please specify)
Additional Information: Supporting documents outlined in NIH agreement.
How to access documents: Data may be requested by emailing amy.janes@nih.gov.
When available: With publication

Additional Information
Who can access the data: Data may be requested by emailing amy.janes@nih.gov. Individuals would need to put a signed data use agreement in place with the NIH in order to potentially receive data and supporting documents.
Types of analyses: Data may be requested by emailing amy.janes@nih.gov for purposes specified in a signed data use agreement with NIH.
Mechanisms of data availability: Data may be requested by emailing amy.janes@nih.gov for purposes specified in a signed data use agreement with NIH.
